# Effects of sentinel lymph node biopsy combined with breast-conserving surgery on surgical-related indexes, serum TPA level and recurrence rate in patients with early breast cancer

**DOI:** 10.12669/pjms.41.3.9863

**Published:** 2025-03

**Authors:** Ke Gong, Siqi Yang, Youzhong Liu, Yong Xu

**Affiliations:** 1 Ke Gong, Department of Thyroid and Breast Surgery, Changde Hospital, Xiangya School of Medicine, Central South University (The First People’s Hospital of Changde City), Changde 415000, Hunan, China; 2 Siqi Yang, Department of Thyroid and Breast Surgery, Changde Hospital, Xiangya School of Medicine, Central South University (The First People’s Hospital of Changde City), Changde 415000, Hunan, China; 3 Youzhong Liu, Department of Thyroid and Breast Surgery, Changde Hospital, Xiangya School of Medicine, Central South University (The First People’s Hospital of Changde City), Changde 415000, Hunan, China; 4 Yong Xu, Department of Thyroid and Breast Surgery, Changde Hospital, Xiangya School of Medicine, Central South University (The First People’s Hospital of Changde City), Changde 415000, Hunan, China

**Keywords:** Breast cancer, Breast-conserving, Prognosis, Sentinel lymph node biopsy, Tissue polypeptide antigen

## Abstract

**Objective:**

To explore the effects of sentinel lymph node biopsy (SLNB) combined with breast-conserving surgery on surgical-related indexes, serum tissue polypeptide antigen (TPA) level and recurrence rate in patients with early breast cancer.

**Methods:**

This was retrospective study. A total of 112 patients with early breast cancer who underwent surgical treatment in The First People’s Hospital of Changde City from January 2020 to January 2024 were enrolled. According to different surgical methods, they were divided into control group (49 cases, modified radical mastectomy) and observation group (63 cases, SLNB combined with breast-conserving surgery). The perioperative indexes, levels of serum tumor markers, postoperative complications, long-term survival and recurrence were compared between the two groups.

**Results:**

The operation time, intraoperative blood loss, postoperative drainage volume, hospitalization time in observation group were significantly lower than those in control group (*P*<0.05). Before surgery and at three months after surgery, there were no significant differences in the levels of serum cancer antigen 153(CA153), carcinoembryonic antigen (CEA), and tissue polypeptide antigen (TPA) between the two groups(*P*>0.05). After three years of follow-up, there were no significant differences in the recurrence rate and survival rate between the two groups (*P*>0.05).

**Conclusion:**

Compared with modified radical mastectomy, SLNB combined with breast-conserving surgery may have better surgical procedures and outcomes, and significantly reduce the incidence of upper limb edema. The effects of surgical method choice are fewer on the long-term prognosis and postoperative levels of tumor markers such as serum TPA.

## INTRODUCTION

Breast cancer has a tendency to deteriorate at a younger age with changes in the environment and people’s living habits. However, up to 10% of patients are still not completely cured even in the early stage of breast cancer, a situation that seriously affects their physical and mental health.^1^,^2^ Recent years have witnessed a marked improvement in early diagnosis and survival rates of breast cancer with advances in tumor screening tools.^3^ Surgery is the preferred method of radical treatment for breast cancer.^4^ Conventionally, radical mastectomy for breast cancer involves the removal of the breast and chest muscles, which is extremely extensive and not applicable to those with breast cancer.

Patients with early breast cancer that does not involve the pectoralis muscle fascia are typically treated with modified radical mastectomy. Postoperative adjuvant chemotherapy is now considered one of the standard treatment modalities for breast cancer.^5^ Sentinel lymph node biopsy (SLNB) combined with breast-conserving surgery, as an emerging radical procedure, has been widely used in developed countries, but has rarely been reported in China.^6^-^8^ For this reason, the study explored the effects of SLNB combined with breast-conserving surgery on surgical-related indexes, serum tissue polypeptide antigen(TPA) level and recurrence rate in patients with early breast cancer.

## METHODS

This was retrospective study. A total of 112 patients with early breast cancer who underwent surgical treatment in The First People’s Hospital of Changde City from January 2020 to January 2024 were enrolled and divided into control group (49 cases, modified radical mastectomy) and observation group (63 cases, SLNB combined with breast-conserving surgery) according to different surgical methods. Patient data including demographic data, diagnosis of breast carcinoma, were retrieved from electronic medical record systems of our hospital.

### Ethics approval:

The study was approved by the Institutional Ethics Committee of the First People’s Hospital of Changde City (No.:2022-257-01; date: November 21, 2022), and written informed consent was obtained from all participants.

### Inclusion criteria:

- Confirmed diagnosis of breast cancer by postoperative pathology.
- TNM Stage-I-II.
- Aged 30~60 years old.
- Having signed the informed consent.

### Exclusion criteria:

- Coagulation disorders.
- Comorbidities with other malignant tumors, allergies, history of axillary surgery.
- Receiving chemotherapy or radiotherapy prior to enrollment.

All patients in both groups were female and no one dropped out of the study midway. Forty-nine cases in the control group were treated with modified radical mastectomy for breast cancer, aged 33-58 years, with a mean age of 46.74±4.28 years, and a mean disease duration of 5.22±2.65 years. As for the tumor location, there were 21 cases in the outer upper quadrant, 12 cases in the inner upper quadrant, 10 cases in the outer lower quadrant, and six cases in the inner lower quadrant; as for the TNM stage, there were 25 cases in Stage-I and 24 cases in Stage-II_A_. Sixty-three cases in the observation group were treated with SLNB combined with breast-conserving surgery for breast cancer, aged 34–62 years, with a mean age of 47.67±4.12 years, and a mean disease duration of 5.02±2.89 years. As for the tumor location, there were 32 cases in the outer upper quadrant, 13 cases in the inner upper quadrant, 12 cases in the outer lower quadrant, and 6 cases in the inner lower quadrant; as for the TNM stage, there were 33 cases in Stage-I and 30 cases in Stage-II_A_. No significant difference was seen in the comparison of the general data of the two groups (*P*>0.05).

### Surgical methods:

### Control group:

Modified radical mastectomy for breast cancer was performed. After general anesthesia, an oblique shuttle incision was selected depending on the location of the lesion, and the tissue around the tumor (>3 cm from the tumor) was excised, conventional lymph node dissection was performed, and the pectoralis major muscle was preserved. Observation group: Sentinel lymph node biopsy (SLNB) combined with breast-conserving surgery was performed. After general anesthesia, a curved incision was selected depending to the location of the tumor, and the tumor and its surrounding (≤2 cm) tissues were excised to ensure the integrity of the breast; intraoperative rapid pathological examination was performed, and those with negative margins could be sutured immediately, while those with positive margins were required to continue to expand the scope of excision until the margins were negative. A 5-cm incision was made at the lower edge of the patient’s axillary hair region to free the flap in the axillary direction, and stained lymphatic vessels and lymph nodes were visualized from the lateral aspect of the pectoralis major muscle parallel to the muscle bundles, and a quick-frozen biopsy was performed when they were located; for positive patients, axillary lymph node clearance was performed.

### Evaluation indexes:

- Comparison of perioperative indexes between the two groups. The operation time, intraoperative blood loss, postoperative drainage volume, and hospitalization time of the two groups were recorded, in which intraoperative and postoperative blood loss were combined with the volume of suction bottle, the quality of gauze, and the area of bloodstain on the surgical bed to be calculated collectively.
- Comparison of serum tumor markers between the two groups. On the day of admission and three months after surgery, 3-5 ml of venous blood was collected from the enrolled patients. After centrifuging, Beckmann AU 5800 Full-automatic Biochemical Analyzer was employed to detect the levels of serum cancer antigen 153 (CA153), carcinoembryonic antigen (CEA), tissue polypeptide antigen (TPA) by ELISA.
- Postoperative complications in both groups. The number of cases of upper limb edema, subcutaneous effusion, and subcutaneous tissue necrosis in both groups after surgery was recorded during hospitalization.
- Comparison of the improvement status of survival quality between the two groups. Before surgery and six months after surgery, the Karnofsky score was evaluated on a scale of 100, with a high score indicating good health. Improved: increase in score >10 points after surgery; Stabilized: increase or decrease in score within 10 points after surgery; Decreased: decrease in score >10 points after surgery. Improvement rate of quality of life = (improved + stabilized)/total cases×100%.
- Postoperative metastasis and recurrence in both groups. The patients were followed up for three years, every three months in the 1st and 2nd years, every six months in the 3rd year, and the last follow-up was in January 2024, with a follow-up rate of 100%. Follow-up visits were primarily carried out in the form of physical examination, routine blood tests, and imaging tests, and recurrence was determined by the presence of new nodules or lumps in the breasts of the patients.The follow-up work of all patients was completed by the same group of surgeons.

### Statistical analysis:

All data were statistically analyzed using the SPSS23.0 software. Enumeration data were expressed as the number of cases (n) and rate (%), and *χ*^2^ test was used for comparisons between the two groups. Measurement data were expressed as mean ± standard deviation (SD), and t test was used for comparison between groups. A *P*-value <0.05 indicated a statistically significant difference.

## RESULTS

The operation time, intraoperative blood loss, postoperative drainage volume, and hospitalization time in the observation group were significantly lower than those in the control group (*P*<0.05), as shown in Table-I.

**Table-I:** Comparison of perioperative indexes between the two groups (*χ̅±S*).

Group	n	Operation time (min)	Intraoperative blood loss (mL)	Postoperative drainage volume (mL)	Hospitalization time (d)
Observation group	63	58.41±21.02	74.45±26.54	108.84±25.98	5.79±2.02
Control group	49	132.22±43.97	128.55±41.68	238.71±41.62	8.62±3.11
*t*		11.723	8.357	20.226	5.818
*P*		<0.001	<0.001	<0.001	<0.001

At three months after surgery, serum CA153, CEA and TPA levels in both groups were significantly lower than those before surgery (*P*<0.05). Before surgery and at three months after surgery, there were no significant differences in the levels of CA153, CEA and TPA between the two groups (*P*>0.05), as shown in Table-II.

**Table-II:** Comparison of serum tumor markers between the two groups (*χ̅±S*).

Group	n	CA153 (U/mL)		CEA (ng/mL)		TPA (U/L)	
		Before surgery	3 months after surgery	Before surgery	3 months after surgery	Before surgery	3 months after surgery
Observation group	63	36.37±4.61	29.85±3.08^①^	11.57±3.01	4.99±1.26^①^	75.77±9.24	52.36±6.81^①^
Control group	49	36.49±3.88	30.02±4.11^①^	11.29±2.84	5.31±1.99^①^	76.15±8.73	53.44±7.22^①^
*t*		0.146	0.250	0.501	1.037	0.221	0.811
*P*		0.884	0.803	0.618	0.302	0.825	0.419

***Note:*** ① : P<0.05 compared with before surgery.

The incidence of upper limb edema in observation group was significantly lower than that in control group (*P*<0.05). No significant difference was seen in the incidence of complications such as subcutaneous effusion and subcutaneous tissue necrosis between the two groups (*P*>0.05), as shown in Table-III.

**Table-III:** Comparison of postoperative complications between the two groups [n (%)].

Group	n	Upper limb edema	Subcutaneous effusion	Subcutaneous tissue necrosis
Observation group	63	1 (1.59)	2 (3.17)	0 (0.00)
Control group	49	5 (10.20)	2 (4.08)	1 (2.04)
*χ* ^2^		4.036	0.066	1.297
*P*		0.045	0.797	0.255

At six months after surgery, improvement rate of Karnofsky score in the observation group was significantly higher than that in the control group (*P*<0.05), as shown in Table-IV. After three years of follow-up, there were no significant differences between the two groups in the recurrence rate and survival rate at one, two, and three years after surgery (*P*>0.05), as shown in Table-V.

**Table-IV:** Comparison of postoperative quality of life between the two groups [n(%)].

Group	n	Improved	Stabilized	Decreased	Overall improvement rate (%)
Observation group	63	38	17	8	55 (87.30)
Control group	49	25	6	18	31 (63.27)
*χ* ^2^					8.933
*P*					0.003

**Table-V:** Postoperative metastasis and recurrence in both groups [n (%)].

Group	n	Postoperative recurrence			Postoperative survival		
		1-year recurrence rate	2-year recurrence rate	3-year recurrence rate	1-year survival rate	2-year survival rate	3-year recurrence rate
Observation group	63	2 (3.17)	4 (6.35)	7 (11.11)	63 (100.00)	62 (98.41)	61 (96.83)
Control group	49	1 (2.04)	3 (6.12)	5 (10.20)	49 (100.00)	49 (100.00)	47 (95.92)
*χ* ^2^		0.136	0.002	0.024	0.000	0.785	0.066
*P*		0.712	0.961	0.787	1.000	0.376	0.797

## DISCUSSION

In this study, the operation time, intraoperative blood loss, and hospitalization time of the observation group were significantly lower than those of the control group, and the improvement rate of quality of life at six months after surgery was also higher than that of the control group, suggesting that patients undergoing SLNB combined with breast-conserving surgery had less intraoperative blood loss, shorter operation time consumed, and faster recovery compared with modified radical mastectomy. This is consistent with the findings of Bargon CA et al.^9^ The reason may be that some patients in the observation group had their upper limb function unaffected because they did not undergo lymph node dissection, coupled with the fact that less breast tissue was removed intraoperatively, leading to a generally faster recovery after surgery.

As one of the secondary sex characteristics of women, breasts are of great importance. Minimizing the change in breast shape from radical mastectomy has been a major concern for both patients and physicians.^10^,^11^ Studies have shown a reduction in the extent of resection in modified radical mastectomy compared to conventional.^12^,^13^ However, it is worth noting that patients will have compromised breast integrity and may be psychologically traumatized due to the greater trauma of the breast tissue removed intraoperatively and the higher number of postoperative complications such as flap necrosis. Also, undifferentiated lymph node dissection may damage the shoulder muscles and reduce the quality of life. Breast conserving surgery has emerged to safeguard breast integrity. Studies concluded that breast-conserving surgery is not dramatically different to radical mastectomy in terms of tumor control and long-term survival, and is more acceptable to patients with obvious advantages.^14^-^16^ Sentinel lymph node biopsy (SLNB), initially used in the treatment of penile cancer, is a minimally invasive surgical procedure aimed at determining the metastatic status of a tumor through the nodes of the sentinel lymph. Sentinel lymph nodes are one or more lymph nodes that drain malignant tumors to the lymph node pool; a positive test result indicates that other lymph nodes may have been invaded by malignant tumors. For breast cancer patients undergoing surgical treatment, a positive axillary sentinel lymph node biopsy on two consecutive occasions indicates the necessity of axillary lymph node dissection for them to reduce the risk of tumor recurrence.^17^,^18^

It was suggested in the study that CA153, CEA, and TPA are three common tumor markers for breast cancer, in which CA153 is highly expressed in a variety of malignant tumor cells; CEA is extremely low in normal cells and is widely used in the diagnosis of adenocarcinomas; TPS can be detected in epithelial malignant tumor cells, with slightly higher diagnostic accuracy for breast cancer recurrence than that of CA153.^19^ In this study, there was no difference between the two groups in terms of serum CA153, CEA, and TPA levels before surgery and three months after surgery, suggesting little effect of different surgical choices on serum tumor marker levels.

The reason may be that the levels of CA153, CEA, and TPA are related to tumor metastasis and invasive ability. Moreover, the patients in this study were in the early stage of breast cancer with poorer invasive ability of tumor cells, so the long-term survival rate was higher after surgical treatment. Furthermore, the incidence of upper limb edema in the observation group was significantly lower than that in the control group, which was consistent with the findings of van Haaren ERM et al.^20^ The reason is that SLNB combined with breast-conserving surgery removes less breast tissue and avoids unnecessary lymph node dissection. With a three years follow-up, we found that there was no difference between the two groups in the recurrence rate and survival rate at one, two, and three years after surgery, suggesting comparable midterm prognostic effects of the two surgical procedures.

### Limitations:

However, there are limitations to the use of this procedure, and advances in medical technology are needed to make breast-conserving surgery more accessible to more patients. While further research is still required as there was a limited sample size in the present study.

## CONCLUSIONS

SLNB combined with breast-conserving surgery results in an effective reduction of intraoperative blood loss, operation time, accelerated postoperative recovery, fewer postoperative complications, and significant improvement of quality of life in patients with early breast cancer. In comparison with modified radical mastectomy, no significant difference is observed in the effects of the two surgical procedures on serum tumor marker levels and three years prognosis of patients.

### Authors’ Contributions:

**KG** and **SY:** Carried out the studies, participated in collecting data, literature search and drafted the manuscript, and are responsible and accountable for the accuracy or integrity of the work.

**YL** and **YX:** Performed the statistical analysis and participated in its design. Critical review.

All authors have read and approved the final manuscript.
